# Analysis of the Use of Cylindrospermopsin and/or Microcystin-Contaminated Water in the Growth, Mineral Content, and Contamination of *Spinacia oleracea* and *Lactuca sativa*

**DOI:** 10.3390/toxins11110624

**Published:** 2019-10-28

**Authors:** Maria Llana-Ruiz-Cabello, Angeles Jos, Ana Cameán, Flavio Oliveira, Aldo Barreiro, Joana Machado, Joana Azevedo, Edgar Pinto, Agostinho Almeida, Alexandre Campos, Vitor Vasconcelos, Marisa Freitas

**Affiliations:** 1 Area of Toxicology, Faculty of Pharmacy, Universidad de Sevilla, Profesor García González n°2, 41012 Seville, Spain; mllana@us.es (M.L.-R.-C.); angelesjos@us.es (A.J.); camean@us.es (A.C.); 2 CIIMAR/CIMAR, Interdisciplinary Centre of Marine and Environmental Research, University of Porto, Terminal de Cruzeiros do Porto de Leixões, Av. General Norton de Matos, s/n, 4450-208 Porto, Portugal; up201510053@fc.up.pt (F.O.); aldo.barreiro@gmail.com (A.B.); joana.ffmachado@gmail.com (J.M.); joana_passo@hotmail.com (J.A.); vmvascon@fc.up.pt (V.V.); 3 LAQV/REQUIMTE, Departament of Chemical Sciences, Faculty of Pharmacy, University of Porto, Rua Jorge de Viterbo Ferreira 228, 4050-313 Porto, Portugal; edgarpinto7@gmail.com (E.P.); aalmeida@ff.up.pt (A.A.); 4 Polytechnic Institute of Porto, Department of Environmental Health, School of Health, CISA/Research Center in Environment and Health, Rua Dr. António Bernardino de Almeida, 400, 4200-072 Porto, Portugal; 5 Biology Department, Faculty of Sciences, University of Porto, Rua do Campo Alegre, s/n, 4169-007 Porto, Portugal

**Keywords:** cyanobacteria, microcystin-LR, cylindrospermopsin, cyanotoxins mixture, plant growth, toxin bioaccumulation

## Abstract

Cyanobacteria and cyanotoxins constitute a serious environmental and human health problem. Moreover, concerns are raised with the use of contaminated water in agriculture and vegetable production as this can lead to food contamination and human exposure to toxins as well as impairment in crop development and productivity. The objective of this work was to assess the susceptibility of two green vegetables, spinach and lettuce, to the cyanotoxins microcystin (MC) and cylindrospermopsin (CYN), individually and in mixture. The study consisted of growing both vegetables in hydroponics, under controlled conditions, for 21 days in nutrient medium doped with MC or CYN at 10 μg/L and 50 μg/L, or CYN/MC mixture at 5 + 5 μg/L and 25 + 25 μg/L. Extracts from *M. aeruginosa* and *C. ovalisporum* were used as sources of toxins. The study revealed growth inhibition of the aerial part (Leaves) in both species when treated with 50µg/L of MC, CYN and CYN/MC mixture. MC showed to be more harmful to plant growth than CYN. Moreover spinach leaves growth was inhibited by both 5 + 5 and 25 + 25 µg/L CYN/MC mixtures, whereas lettuce leaves growth was inhibited only by 25 + 25 µg/L CYN/MC mixture. Overall, growth data evidence increased sensitivity of spinach to cyanotoxins in comparison to lettuce. On the other hand, plants exposed to CYN/MC mixture showed differential accumulation of CYN and MC. In addition, CYN, but not MC, was translocated from the roots to the leaves. CYN and MC affected the levels of minerals particularly in plant roots. The elements most affected were Ca, K and Mg. However, in leaves K was the mineral that was affected by exposure to cyanotoxins.

## 1. Introduction

Cyanobacteria are anaerobic photoautotrophic group of primitive microorganisms widely distributed in freshwater [1]. The overgrowth of cyanobacteria is favored by several factors related with the eutrophication of aquatic ecosystems, which has increased in the last decades due to the intensification of agricultural and industrial activities, and also with climate change [1,2]. The concerns raised about the cyanobacteria are related with the cyanotoxins that many of these species and strains produce. Among the most prevalent cyanotoxins found in freshwaters are microcystins (MCs). MCs are cyclic heptapeptides and more than 200 chemical variants have been described so far [3]. Moreover they are potent tumor promoters and carcinogenic compounds [4]. Microcystin-LR (MC-LR, herein referred as MC), with leucine and arginine respectively in the positions X and Z of the molecule common structure is the most toxic variant known. The main mechanism of toxicity of these compounds is the specific binding and inhibition of protein phosphatases [5,6,7]. In fresh waters MCs concentrations can reach values from 108 µg/L up to 10,000 µg/L [8,9,10], however, the most common concentration range of MCs in surface and irrigation waters vary from 4–50 µg/L [11].

Cylindrospermopsin (CYN) is a tricyclic alkaloid with the following chemical formula: C_15_H_21_N_5_O_7_S. CYN has been associated with kidney toxicity and liver failure. CYN is also referred to display genotoxic activity [12,13,14,15]. Furthermore the toxin has been recognized as a nationwide threat due to the invasive nature of its main producer, *Cylindrospermopsis raciborskii* [16]. At the molecular level CYN may interact with the ribosomes and inhibit the synthesis of proteins in the cells [17]. CYN has been reported in surface waters at concentrations up to 173 µg/L [1] and also to co-occur with MCs [18].

The main route of human exposure of these cyanotoxins is the consumption of contaminated water or fish. However, the consumption of contaminated vegetables and food supplements may also contribute as an important source of human exposure [19]. Indeed vegetables are potential repositories of cyanotoxins when irrigated with contaminated freshwater [20]. This agricultural practice is potentially detrimental to plant development, plant yield and quality. MCs have been shown to affect root and shoot development, to inhibit seed germination and to affect plant metabolism [21,22,23,24]. Changes in photosynthesis, alterations in activity of oxidative stress defense enzymes, increase in lipid peroxidation and accumulation of reactive oxygen species (ROS) have also been attributed to exposure to MCs [2,25,26,27].

CYN also has shown to be adverse to plants, especially at high concentrations in the water (above 100 µg/L) [28,29,30]. CYN can cause oxidative stress [31]; inhibit germination [28] and growth [32,33] and trigger a range of biochemical effects [34]. Recently, programed cell death symptoms were reported in two model vascular plants after exposure to CYN 100 µg/L [35]. Contrarily, exposure to low concentrations of CYN (below 100 µg/L) might not be detrimental to plants. For instance, absence of effects, or even stimulation of growth, were reported by Freitas et al. (2015) [30] in hydroponic cultures of lettuce exposed 1 or 10 µg/l MC, CYN or MC+CYN [30], and by Machado et al. (2017) [36] in soil grown carrot plants irrigated for 1 month with 50 µg/L MC. In addition, Guzman-Guillén et al. (2017) [37] reported increased growth of carrots exposed to 50 µg/L CYN, and aromatic plants like parsley (*Petroselinum crispum* L.) and coriander (*Coriandrum sativum* L.) also showed tolerance to MC and CYN [38].

Alongside with the impairment of growth, MCs have shown to alter other plant traits of agronomic relevance and to affect nutritional parameters in cultivated plants. Freitas et al. (2015) [30] showed that cyanotoxins such as MC and CYN interfere with mineral accumulation in lettuce. Moreover, changes in the metabolism of plants exposed to cyanotoxins were also suggested to lead to the accumulation of potential allergenic proteins [39]. The exposure to MC could also be a plausible cause of the decrease in the accumulation of ascorbic acid (vitamin c) in this root vegetable [36].

El Khalloufi et al. (2016) [40] and Lahrouni et al. (2016) [41] showed that toxic cyanobacterial extracts containing MC affect plant-bacterial symbioses and decrease the bacterial growth rate of rhizospheric microbiota. Leguminous crops that develop symbiotic interactions with *Rhizobia* to uptake nitrogen can be particularly affected in such growth conditions. For instance, the chronic exposure of fava beans to contaminated water (50 and 100 µg/L MC) was shown to reduce significantly plant-Rhizobia nodule number and nitrogen assimilation (measured as dry matter) in the plants, as well as plant growth and photosynthetic activity [42].

Although a provisional upper limit in drinking water of 1 µg/L for MC has been proposed by the World Health Organization (WHO); and the United States Environmental Protection Agency (US-EPA) has set a health advisory for CYN of 3 µg/L, in general, there are very few countries with legislation concerning cyanotoxin levels in food. The legislation is addressed mainly to fish and shellfish products but does not cover vegetables or other food products and supplements [43]. The lack of legislation is probably a consequence of the lack of knowledge concerning the contamination and the impact of cyanotoxins in the quality and safety of vegetables. For instance, more than one type of cyanotoxin is frequently detected in the environment, nevertheless the bioactivity and toxicology of such combinations in plants has been overlooked. Moreover, the genetics may play a critical role in the sensitivity of plants to cyanotoxins. This factor has also been poorly considered in research, and there is a general lack of understanding of the susceptibility of plant crops to cyanotoxins and the genetic factors that determine the susceptible phenotype. This work aim to cover some of the knowledge gaps in this field of research, namely the susceptibility of green-vegetables to cyanotoxins at environmentally relevant concentrations, and cyanotoxins accumulation in plants. In the present work *Spinacea oleracea* and *Lactuca sativa*, two common vegetables used in the human diet, were exposed to environmentally relevant concentrations (10 and 50 µg/L) of MC and CYN, or their mixtures, through crude extracts of *M. aeruginosa* and *C. ovalisporum*, respectively. The effects of these cyanotoxins on growth, photosynthesis, mineral content and bioaccumulation on spinach and lettuce are here reported and discussed in relation to susceptibility to cyanotoxins, nutritional value, food safety, and human exposure.

## 2. Results and Discussion

### 2.1. Effects of CYN, MC and CYN/MC Mixture on Spinach and Lettuce Growth

**Figure 1:**
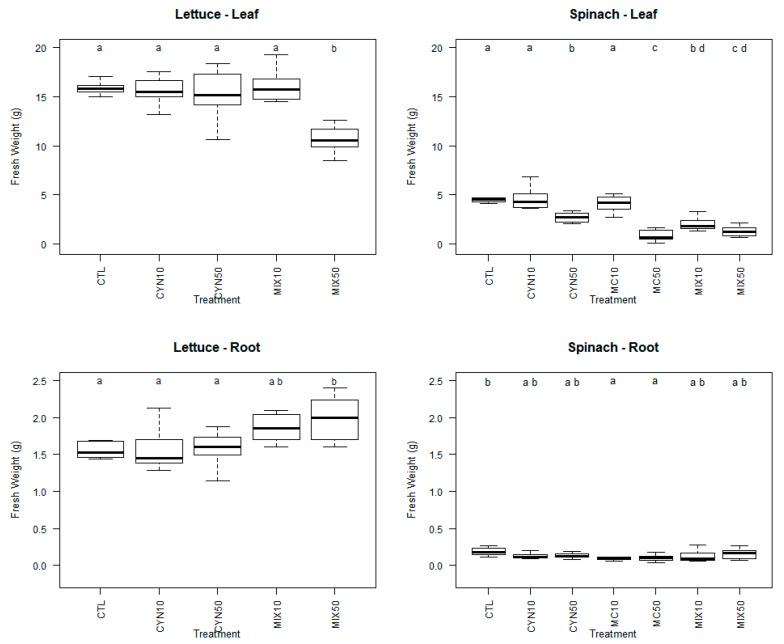
Box-whiskers plots of fresh weight of spinach and lettuce plants (leaves and roots) exposed to cylindrospermopsin (CYN) and cyanotoxins microcystin (MC) at the concentrations of 10 µg/L (CYN10 and MC10) and 50 µg/L (CYN50 and MC50) and CYN/MC mixture at the concentrations of 5 + 5 µg/L (MIX10) and 25 + 25 µg/L (MIX50), respectively, for 21 days. Different letters (**a**–**d**) mean significant differences (*p* < 0.05). Control plants (CTL). Number of sample replicates (n = 8).

The effects of CYN, MC and CYN/MC mixture on spinach and lettuce growth were studied by comparing the fresh weight of the control and treated plants at different environmental concentrations (CYN and MC: 10 and 50 µg/L; CYN/MC mixture: 5 + 5 µg/L and 25 + 25 µg/L, respectively) (Figure 1).

**Figure 2:**
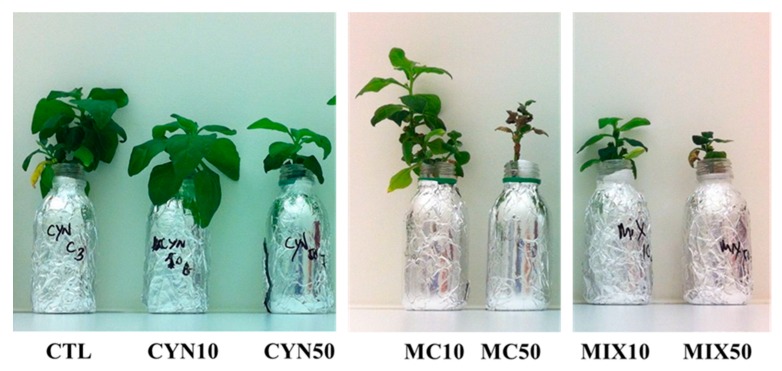
Representative images of spinach plants grown for 21 days with CYN and MC at the concentrations of 10 µg/L (CYN10 and MC10) and 50 µg/L (CYN50 and MC50) and CYN/MC mixture at the concentrations of 5 + 5 µg/L (MIX10) and 25 + 25 µg/L (MIX50), respectively, for 21 days. Control plants (CTL).

In general, for all exposure conditions, the response at the physiological level was concentration-dependent and more noticeable decreased growth was observed for spinach plants (roots and leaves). The results also show that spinach plants were more vulnerable to MC than CYN and this was also verified at morphological level, once for the treatment applied with MC 50 µg/L spinach plants were negatively affected resulting in mortality and deleterious effects in leaves (necrosis) (Figure 2). Decreased growth caused by MC on terrestrial plants was already reported in previous studies [21,23,26,44,45,46,47,48]. However, most of them were conducted under unrealistic conditions, where plants were exposed to quantities 100–1000-fold above the environmentally realistic concentrations. Nevertheless, recent studies in which exposure experiments were carried out with environmentally realistic concentrations (5 µg/L and 10 µg/L) have shown that depending on the plant species, MC can negatively affect plant productivity and quality, being the edible fraction reduced by the MC exposure [49]. Some examples can be enumerated: (1) lettuce plants treated with 5 and 10 μg/L MC were shorter, had fewer leaves per head and weighed less than the control group [49]; (2) carrot plants exposed to 1, 5, 10 μg/L MC decreased the total mass of the head and diameter of the roots relative to the control group [49]; (3) green beans treated with 1, 5, 10 μg/L MC had lower total mass and fewer beans per plant than the control [49]; and (4) the growth of cucumber at different growth stages was inhibited with exposure to 10 μg/L MC, and the order of growth inhibition was seedling stage > early flowering stage > fruiting stage [50].

However, contrarily, some studies showed no effects in crop plants due to MC exposure at environmentally realistic levels. Cao et al. (2018) [51] reported that irrigation with natural concentrations of MC-contaminated water did not affect the growth and yield of lettuce and rice. Liang et al. (2016) [52] also reported that low concentration of MCs (1 µg/L) did not affect the growth and photosynthesis of rice. Furthermore, interestingly, the productivity and nutritional quality of some agricultural plants may even be enhanced when exposed to ecologically relevant concentrations of MCs, with a weight of leaves increasing, as it was shown in tomato and lettuce plants, respectively [30,53]. In this study, the results obtained for spinach exposed to MC corroborate the findings of Pflugmacher et al. (2007) [26], in which the exposure of different variants of spinach to cyanobacterial crude extract containing 0.5 μg/L MC, resulted for some variants, in the reduction of growth due to the lower leaf size and the lower number of leaves than in control groups. In line with the study of Pflugmacher et al. (2007) [26], here we report differences in the sensitivity of the two vegetables (spinach and lettuce) to CYN, which shows that sensitivity to cyanotoxins is, in part, determined by genetic factors. Despite the differences in the experimental designs and although the toxicity mechanisms involved remain unknown, together, these results raise attention for species that seem to be most vulnerable to adverse effects of MC exposure, which should be considered by the regulatory agencies and policy-makers when outlining legislation for irrigation water.

Less is known about the potential effects of CYN and CYN/MC mixture on productivity and quality of crop plants. In this work, spinach plants showed to be more sensitive to environmentally realistic concentrations of cyanotoxins than lettuce plants. The exposure of spinach plants to CYN led to a significant decrease in the fresh weight of leaves at the highest concentration tested (50 µg/L) (*p* < 0.05) (although lower than for MC at the same concentration). For lettuce, plants it is important to note that CYN at 10 and 50 µg/L did not result in significant differences in root and leaf weight compared to control group. Previous studies have shown that although low concentrations of CYN (1–10 µg/L) can stimulate the root and leaf growth, the exposure of lettuce plants to 100 µg/L CYN negatively affected the leaf yield by the reduction of its fresh weight [30]. So, as the exposure conditions were similar in both studies, this can be indicative that lettuce plants are able to tolerate CYN at least till concentrations of 50 µg/L without deleterious effects on their productivity. Data also exists for rice plants which were exposed to 2.5 µg/L CYN, resulting in a significant increase in root fresh weight upon 48 h of exposure and a significant decrease in leaf fresh weight of leaves after nine days of exposure [31].

Regarding the cyanotoxins mixture, interestingly, only the fresh weight of spinach leaves was significantly lower than the control group (*p* < 0.05), being the effect more prominent for the highest concentration. For lettuce, a significant increase in fresh weight of roots and a significant decrease in fresh weight of leaves was only observed for plants exposed to CYN/MC mixture at the highest concentration (25 + 25 µg/L CYN/MC) (*p* < 0.05). Our results are in agreement with those reported by Freitas et al. (2015) [30], which have suggested that lettuce plants are able to cope with low concentrations (1 and 10 μg/L) of CYN and CYN/MC mixture by ensuring the maintenance of and even increasing their fresh weight. Prieto et al. (2011) [31] have studied the interaction effects of CYN (0.13 µg/L) and MC (50 µg/L), on rice plants and the mixture did not produce significant changes in fresh and dry weight of roots and leaves after 48 h of exposure. In our study, it can be hypothesized that the increased metabolic activity in roots required to maintain or improve the growth could compromise the leaf growth of the spinach and lettuce plants, respectively. It is also important to point out that, in this study, when plants were exposed to CYN/MC mixtures, even in concentrations that individually plants seem to be susceptible of homeostatic compensation (e.g., 25 µg/L), the effects observed were exacerbated. 

Collectively, these results demonstrate that MC can severely inhibit the growth and performance of spinach plants, and that the combined effect of CYN and MC was more intense than their individual effects for lettuce plants. This suggests that the presence of multiple cyanotoxins in irrigation water, even at environmentally realistic concentrations may influence the productivity of crop plants in a species-dependent manner. As the simultaneous presence of multiple cyanotoxins in aquatic ecosystems is frequent, being MC the most prevalent cyanotoxin and CYN has been increasingly recurrent [11], the potential interaction between the mechanisms of toxicity of CYN (inhibition of protein synthesis) [54,55] and MC (inhibition of serine/threonine PP) [56,57] should be further studied at cellular level in plants.

### 2.2. Effects of CYN, MC and CYN/MC Mixture on Lettuce and Spinach Photosynthetic Capacity

**Figure 3:**
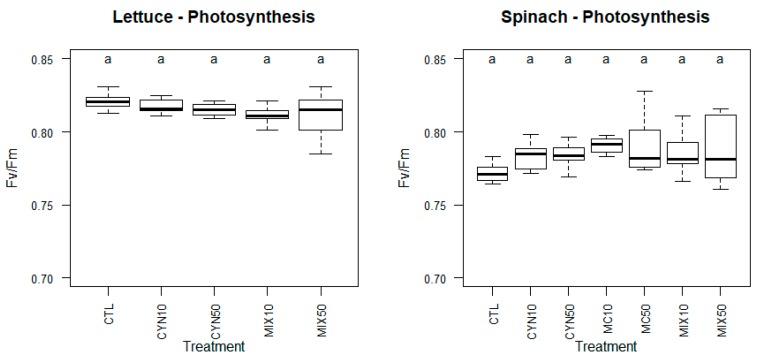
Box-whiskers plots of maximum fluorescence (Fv/Fm) of spinach and lettuce plants exposed CYN and MC at the concentrations of 10 µg/L (CYN10 and MC10) and 50 µg/L (CYN50 and MC50) and CYN/MC mixture at the concentrations of 5 + 5 µg/L (MIX10) and 25 + 25 µg/L (MIX50), respectively, for 21 days. Different letters (a, b, c and d) mean significant differences (*p* < 0.05). Control plants (CTL). Number of sample replicates (n = 8).

The treatment with 50 µg/L MC or with the toxin mixture, as reported in Figure 1 and Figure 2, was highly toxic to spinach, leading to extended leaf necrosis and even to the death of some plants. Thereby measurements of chlorophyll fluorescence were carried out only in plants that survived those treatments. In non-stressed plants Fv/Fm values are known to vary between 0.75 and 0.85 [58]. As reported in Figure 3 all Fv/Fm values for the spinach plants analyzed were above 0.76 meaning that chlorophyll fluorescence and the photosynthetic capacity of spinach plants was not affected by treatments with MC and CYN toxins. Of notice is, nevertheless, the increased variability of Fv/Fm values in the 50 µg/L MC and 25 + 25 µg/L CYN/MC treatments, which might evidence some adverse effects of the cyanobacterial extracts in particular spinach plants. Indeed, some of spinach plants in 50 µg/L MC and 25 + 25 µg/L CYN/MC groups evidenced necrotic symptoms in leaves and reduced growth. 

Maximum fluorescence of lettuce plants was not affected by any of the treatments and all values reported for lettuce were above 0.76. Maximum fluorescence yield is directly related to reaction center II (PSII) function. Moreover the decrease of this parameter has been attributed to PSII malfunction, or inhibition of electron transport linked with excitation of reaction centers [59]. A variety of effects of cyanotoxins on plant photosynthesis have been reported. Inhibition of photosynthesis has been consistently reported in plants exposed to high concentrations (usually above 100 µg/L) of MC. Freitas et al. (2015) [39], in a proteomics study in lettuce provided insights regarding the putative molecular events that could be causing the inhibition of photosynthesis by CYN and MC. The authors reported alterations in the expression of key proteins associated with primary photosynthesis reactions (light reactions) such as quinone oxidoreductase, oxygen-evolving enhancer proteins, chlorophyll a-b-binding proteins, chloroplast PsbO4, cytochrome b6/f heme-binding protein 2 and ferredoxin-NADP reductase, but also proteins involved in the Calvin cycle (carbon fixation reactions), ribulose-1,5-bisphosphate carboxylase/oxygenase activase (RuBPactivase), ribulose bisphosphate carboxylase/oxygenase activase 1 (RuBisCO activase 1), phosphoribulokinase (PRK), and sedoheptulose-1,7-bisphosphatase (SBPase), photorespiration (gamma carbonic anhydrase-like 2).

On the other hand, contrasting effects have been reported in studies with toxin concentrations below 100 µg/L. While in tomato and *Vicia faba* the exposure to respectively 100 µg/L MC and 50–100 µg/L MC resulted in a decrease of Fv/Fm [42,60], in carrots for instance, the exposure to water contaminated with 10–50 µg/L MC and 50 µg/L CYN [36] resulted in an increase in the maximum fluorescence yield in this root vegetable. The increase in Fv/Fm after exposure to cyanotoxins was interpreted by Machado et al. (2017) [36] as a plant mechanism of defense related with the enhancement of the physiological condition through improving the photosynthetic capacity. Also Bittencourt-Oliveira et al. (2016) [61] related the increase in the net photosynthetic rate in lettuce plants exposed to *M. aeruginosa* toxic extracts (0.65 to 13 μg/L total MC) as a mechanism of lettuce plants to synthesize additional substrates to supply energy and to be utilized in the biosynthesis of important antioxidant molecules and enzymes that protect from oxidative stress induced by MC. The presented results are thus consistent with previous studies and show that MC or CYN, in the range of 10–50 µg/L, is not detrimental to plant photosynthesis.

### 2.3. Effects of CYN, MC and CYN/MC Mixture on Lettuce and Spinach Mineral Content

Essential mineral elements are usually classified as macronutrients or micronutrients, according to their relative concentration in plant tissue [62]. In this study, the content of macronutrients (Ca, Mg and K) and micronutrients (Mn, Na, Cu, Zn and Fe) was determined in roots and leaves of spinach and lettuce plants exposed to CYN, MC and CYN/MC mixture, in order to assess the physiological condition and nutritional quality of the edible parts of these two vegetables.

#### 2.3.1. Macronutrients

**Figure 4:**
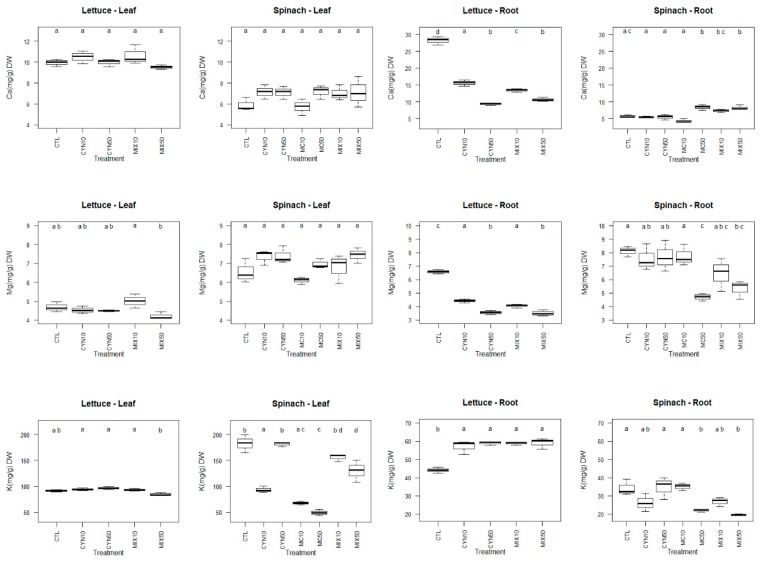
Box-whiskers plots of macronutrients (Ca, Mg and K) content of spinach and lettuce plants (leaves and roots) exposed to CYN and MC at the concentrations of 10 µg/L (CYN10 and MC10) and 50 µg/L (CYN50 and MC50) and CYN/MC mixture at the concentrations of 5 + 5 µg/L (MIX10) and 25 + 25 µg/L (MIX50), respectively, for 21 days. Different letters (a, b, c and d) mean significant differences (*p* < 0.05). Control plants (CTL). Number of sample replicates (n = 3).

The effects of CYN, MC and CYN/MC mixture on the macronutrients content in spinach and lettuce plants (roots and leaves) are presented in Figure 4.

Overall, the detrimental effects of exposure to CYN, MC and CYN/MC mixture were more pronounced in roots than in leaves of spinach and lettuce plants. The results show that there were no significant differences in Ca and Mg content in the edible portion (leaves) of spinach and lettuce plants exposed to CYN, MC and CYN/MC mixture in comparison to the respective control groups. The exception was for K, which content was significantly decreased in spinach leaves (*p* < 0.05) in several exposure conditions, being MC at both exposure concentrations (10 µg/L and 50 µg/L) the most deleterious toxin. Indeed, some studies have demonstrated that MC at ecologically realistic concentrations promotes negative effects in mineral content of leaf plants, generally higher than CYN and MC/CYN mixture. Regarding lettuce, it was reported that exposure to different concentrations of MC (1, 10 and 100 µg/L) resulted in a decrease of the leaf mineral content, and the effects were more pronounced at the highest time and concentration of exposure [30]. In addition, the content of K and Ca in the shoots of *V. faba* have been reported to decrease after two months of exposure to *M. aeruginosa* extract containing 50 and 100 µg/L of MCs [42].

The effects of CYN and CYN/MC exposure on the mineral content in plants have been studied to a much lesser extent than MC. According to our knowledge, this is the first study reporting the effects of CYN and CYN/MC mixture on the mineral content in spinach plants. For lettuce leaves, Freitas et al. (2015) [30] stated similar results to those obtained in this study, with no significant effects on Ca, Mg and K content after 10 days of exposure to CYN and CYN/MC mixture. However, it should be highlighted the tendency for the decreasing in Mg and K content in the lettuce leaves at the highest concentration of CYN/MC mixture. For spinach, interestingly, despite the significant decrease in K content after plant exposure to CYN 10 µg/L and to the mixture at both concentrations (*p* < 0.05), the effects were less deleterious than for MC alone (at both exposure concentrations).

More complex responses were obtained for the mineral content in roots of spinach and lettuce plants. The roots of spinach plants were significantly affected by 50 µg/L MC and CYN/MC mainly at the highest concentration of exposure, resulting in a significant increase in Ca, and significant decreases in Mg and K contents in roots, compared to control group (*p* < 0.05). Similarly to the range of concentrations used in this study, the K in the roots of *V. faba* were decreased, after two months of exposure to *M. aeruginosa* extract containing 50 µg/L MC [42]. However, the responses of spinach roots were not parallel with those obtained for carrot roots at the same concentrations of exposure (10 and 50 µg/L MC) [36], being even contrary for Mg and K (significant increase in carrots and decrease in spinach). In lettuce, in this study, it was also obtained a significant increase in K and a significant decrease in Ca and Mg content in roots in all exposure conditions (CYN and CYN/MC), being the effects more pronounced at the highest concentrations (*p* < 0.05). Similar results for K content in roots were also obtained for *Lycopersicon esculentum* [24].

These results show that the physiological response and consequently the changes in roots mineral content may vary according to the plant species. This was already suggested by Saqrane et al. (2009) [46], which found a concentration-dependent increase in the macro mineral content (Na, K, Ca, P, and N) in roots of *Triticum durum, Zea mays, Pisum sativum, Lens esculenta* after plants exposure to MC. Guzmán-Guillén et al. (2017) [37] also suggest that carrots, a root vegetable, seem to be more vulnerable to CYN (in terms of mineral content) than leafy vegetables, such as lettuce plants. The results obtained for spinach (roots) are in agreement with that suggestion, once for the concentrations tested (10 and 50 µg CYN/L) there were no changes in Ca, Mg and K content in comparison to the control group. However, in this study, the content of Ca and Mg in roots of lettuce plants followed a pattern similar to the one reported in carrots [36]. Lahrouni et al. (2013) [42] proposed that the changes in minerals might result from changes of plant membrane permeability caused by cyanotoxins such as MC. Furthermore, the antioxidant response to stress promoted by cyanotoxins is usually more pronounced in roots than in leaves of exposed plants [30,31]. In fact, since roots are the part of the plant in direct contact with cyanotoxins, it is understood that the uptake of nutrients in spinach and lettuce plants may have been considerably affected by oxidative stress, cellular damage and changes in membrane permeability caused by the toxins. Minerals are essential to plant development since they are intrinsic components of all cellular structures and molecules, and key-players in the metabolism and function of plants [62]. On the other hand, imbalances in a specific element may induce deficiency or excessive accumulation of another element [62]. Although each essential element participates in many different metabolic reactions, it is possible to anticipate which general functions of plant metabolism will be affected due to the disturbance of mineral uptake and translocation promoted by extracts of CYN, MC and CYN/MC. 

Correspondingly, it is well known that Mg and K content in plants can significantly affect photosynthesis and K, which is required as a cofactor for more than 40 enzymes, is the principal cation in establishing cell turgor and maintaining cell electroneutrality of plant cells [62]. Mg, besides being part of the ring structure of the chlorophyll molecule, also has a specific role in the activation of enzymes involved in respiration, photosynthesis and the synthesis of DNA and RNA [63]; and Ca is required for the normal functioning of plant membranes and has been implicated as a second messenger in plant responses to both environmental and hormonal stimuli (calcium acts as a signal to regulate key enzymes in the cytosol) [62]. It is also used in the synthesis of new cell walls and in the mitotic spindle during cell division [62].

The ability of crop plants to cope with abiotic stress, maintaining or even increasing the nutritional value is of utmost importance for food security. In this study, the mineral content (Ca, Mg and K) of the edible parts of lettuce and spinach plants were almost not changed in comparison to the control group (except for K in spinach plants), which enables to conclude that, at least at this level, lettuce plants are able to maintain its mineral content if exposed till 50 µg/L of CYN and MC. Nevertheless, the effects of the CYN/MC mixtures should still be studied in greater detail.

#### 2.3.2. Micronutrients

**Figure 5:**
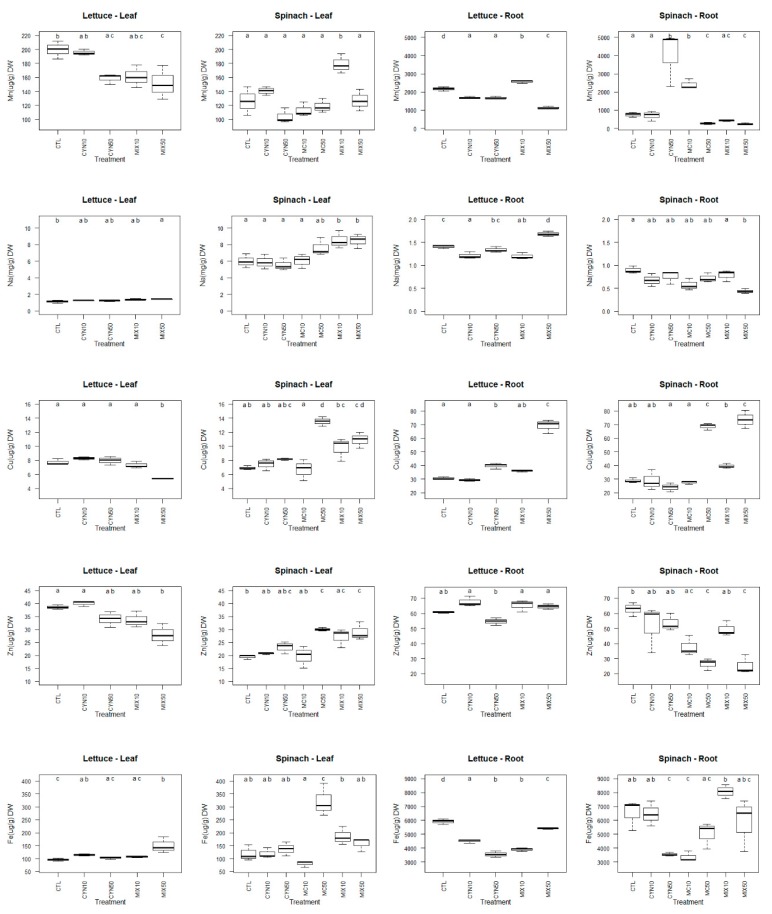
Box-whiskers plots of micronutrients (Mn, Na, Cu, Zn and Fe) content of spinach and lettuce plants (leaves and roots) exposed to CYN and MC at the concentrations of 10 µg/L (CYN10 and MC10) and 50 µg/L (CYN50 and MC50) and CYN/MC mixture at the concentrations of 5 + 5 µg/L (MIX10) and 25 + 25 µg/L (MIX50), respectively, for 21 days. Different letters (**a**–**d**) mean significant differences (*p* < 0.05). Control plants (CTL). Number of sample replicates (n = 3).

The impact of cyanotoxins on micronutrients content in crop plants has been studied to a less extent than macronutrients [24,42,63]. The effects of CYN, MC and CYN/MC mixture on the micronutrients content in spinach and lettuce plants (roots and leaves) are presented in Figure 5.

In general, the changes in micronutrients content were more pronounced in roots than in leaves of lettuce and spinach plants and the effects of CYN/MC mixture were greater than the single cyanotoxins. In lettuce leaves, comparatively to the control group, the content in Mn, Cu and Zn was significantly decreased and the content in Na and Fe was significantly increased, mainly due to the exposure to the MC/CYN mixture at the highest concentration (25 + 25 µg/L). The results obtained for Mn and Zn are in agreement with those reported by Freitas et al. (2015) [30], in which the mixture of MC/CYN (100 + 100 µg/L) led to a significant decrease of their content in lettuce leaves. However, being the same plant species (*Lactuca sativa*) and the experimental conditions similar, the content of Cu in leaves, followed a contrary tendency when compared to the results of Freitas et al. (2015) [30]. Although speculative, a possible explanation can be related with differences of the use of purified cyanotoxins vs. cyanobacterial crude extracts. Thus, it can be hypothesized that the crude extracts may have some substances that function as Cu chelators. Furthermore, in the study developed by Freitas et al. (2015) [30], the mineral content significantly increased in lettuce leaves due to the CYN exposure, and apparently these effects were time and concentration-dependent. However, in this study, in general, there were no significant differences between the content in micronutrients in lettuce leaves exposed to CYN (10 and 50 µg/L) and the control group. 

Regarding spinach leaves, overall, the content of all micronutrients analyzed (Mn, Na, Cu, Zn, Fe) was significantly increased; and most of the changes occurred for the conditions MC 50 µg/L and MC/CYN (25 + 25 µg/L). The result achieved for Na content in spinach leaves is corroborated with that obtained for shoots of *V. faba*, in which plants treated with 50 and 100 μg/L MC significantly increased its content [42].

Concerning the micronutrient content in roots, once again, the results suggest that although lettuce and spinach plants were affected by cyanotoxins, the susceptibility to the different conditions seems to be dependent of the plant species and genetic factors. Lettuce plants were especially affected by CYN/MC mixture at the highest concentration of exposure, while spinach plants were affected by MC 50 µg/L at the similar extent of CYN/MC mixture at the highest concentration of exposure. Overall, due to 25 + 25 µg/L CYN/MC exposure, the lettuce roots significantly increased its content in Na and Cu, significantly decreased its content in Mn and Fe and no changes were registered for Zn content in comparison to the control group. Furthermore, the exposure of lettuce plants to CYN resulted in a significant decrease in Mn and Cu content in roots. Contrasting to our results, Guzmán-Guillén et al. (2017) [37] reported that only Mn and Cu were significantly increased in roots of *Daucus carota* after exposure (30 days) to CYN (10 and 50 µg/L).

The spinach roots showed a significant increase in Cu and a significant decrease in Mn, Na and Zn content when compared to the control group due the exposure to MC 50µg/L and 25 + 25 µg/L CYN/MC. Again, contrary to our results, a significantly higher content of Na in roots of *Triticum durum, Zea mays, Pisum sativum*, *Lens esculenta* [46], *L. esculentum* [24], *V. faba* [42] and *Daucus carota* [36] was found after a prolonged exposure to MC. The uptake of minerals by the plant, especially micronutrients, can be affected by various factors. Thus, the contradictory results can be attributable to different plant species, different time and concentrations of exposure, use of purified toxins or crude extracts, different mechanisms of enzymatic and non-enzymatic defense system and the systems where plants grow (hydroponic vs. soil-grown systems).

Inadequate (deficit or excessive) uptake of micronutrients by crop plants can result in physiological disorders, which may have implications in its antioxidant defense system, yield and nutritional quality. The biological functions which could be affected by micronutrients imbalances comprises antioxidant activity and cell growth. Fe and Cu are associated with enzymes involved in redox reactions; and Na is involved in the synthesis of new cell walls, cell division and cell expansion [62]. Mn is required for activity of some dehydrogenases, decarboxylases, kinases, oxidases, and peroxidases, playing an important role in the structure of photosynthetic proteins as well as ATP synthesis [62]. In addition, Zn is required for many enzymes and also for chlorophyll biosynthesis [62].

Finally, although the enhancement of mineral content in the edible parts of plants is of utmost importance for its nutritional quality, it can result in unintended consequences for public health due to the potential accumulation of cyanotoxins in edible tissues. Furthermore, it is important to point out that the assays were performed in controlled environmental condition, but the changes in mineral nutrition can predispose more the plants to diseases, which can be of concern due to the actual threatens of climate change.

### 2.4. Accumulation of CYN and MC in Plant Leaves

The mean CYN concentration found in the leaves of lettuce exposed to the toxin, at 10 and 50 µg/L CYN, were 2.4 and 9.4 µg CYN/kg fw (Table 1), respectively, determined with the quantification method described by Prieto et al. (2018) [64]. This means that the total amount of CYN taken-up, in relation to the amount present in the culture medium, varied between 23.7% and 18.8%, depending on the CYN concentration tested, and globally it could be considered around 20%. For spinach, in the same exposure conditions, higher concentrations of CYN (9.52 and 36.97 µg/kg fw, respectively) were detected (Table 1). MC was not detected at any concentration tested. For CYN-exposed spinach, the uptake of plant leaves was 95% and 74%, for the exposure to 10 and 50 µg/L CYN, respectively. These results evidence that spinach leaves accumulate more CYN than lettuce, and this might be related with the mechanisms of transport of molecules in the plant tissues which might require specific molecular transporters. The increased CYN accumulation coincided with the inhibition of growth of spinach, being thereby one of the plausible causes due to the toxic effects of CYN in spinach tissues.

Regarding the exposure to toxins mixture, data of CYN and MC in lettuce and spinach plants were already reported previously, in a work aiming to validate a new method for their simultaneous determination in vegetable matrices [18]. In the present work, results from the uptake of MC and CYN are again presented, aiming at a more detailed discussion of results in the context of the plant response and CYN and MC phytotoxicity (Table 1). The two toxins were analyzed in roots and leaves of plants exposed to CYN/MC mixtures at the concentrations of 5 + 5 µg/L and 25 + 25 µg/L. The accumulation of MC in roots of both vegetables increased concomitantly with the increase of concentration of exposure. No significant differences in the accumulation of MC were observed between the two vegetables (Table 1). MC content varied between 0.22 and 0.53 µg/kg fw in 5 + 5 µg/L CYN/MC group and between 1.10 and 1.31 µg/kg fw in 25 + 25 µg/L CYN/MC group. In contrast no MC was detected in leaves of both vegetables.

CYN accumulated at much higher levels than MC in both plant species. Moreover, CYN was detected in leaves as well as in roots of both plant species (Table 1). The accumulation of CYN increased with the increase in the concentration of exposure, in most of the tissues analyzed except in the roots of spinach. Moreover, in lettuce CYN accumulated more in roots than in leaves. These differences were not so evident in spinach and the highest accumulation was observed in the leaves of plants exposed to 25 + 25 µg/L CYN/MC. Mean CYN content estimated in leaves of lettuce and spinach exposed to 5 + 5 µg/L CYN/MC and 25 + 25 µg/L CYN/MC were respectively 10.0–41.9 µg/kg fw and 12.6–119.7 µg/kg fw. In lettuce roots, mean CYN content varied between 34.2 and 110.0 µg/kg fw, whilst in spinach between 39.2 and 24.0 µg/kg fw. These results evidence that CYN is more easily taken-up by the roots than MC. In contrast to MC, CYN was translocated from the roots to the leaves meaning that this toxin can be transported through the plant vascular system. The patterns of accumulation of CYN and MC in plant tissues might be related to differences in their chemical structures and properties and the mechanisms of transport. MC is rather hydrophobic and also possess polar functions [65], therefore the transport of the molecule into the cells requires specific membrane transporters. In animal cells the molecule is known to be transported specifically by OATP type membrane transporters [66] but in plant cells the mechanism of transport is unknown. CYN has approximately half of molecular mass of MC and is highly soluble in water, thus being more easily transported than MC.

Overall the results show that the detrimental effects of cyanotoxins in lettuce and spinach are related with the accumulation of these compounds in the plant tissues. Despite the absence of accumulation in leaves, MC proved to affect severely leaf development, but causes no effect or may even contribute to the stimulation of root development. CYN, on the other hand, is accumulated at higher levels in leaves and roots of spinach and lettuce; however, it seems to be less detrimental to plant development than MC, having lower effects on fresh weight and Fv/Fm. Nevertheless the increased accumulation of CYN in spinach leaves, in comparison to lettuce leaves, could in part explain the increased toxicity and inhibition of growth observed in spinach exposed to CYN50, MIX10 and MIX50.

Studies of MC accumulation in plants suggest that shoot translocation of the toxin may be dependent on plant species and exposure conditions (toxin concentration, time of exposure). Crush et al. (2008) [67] did not detect toxin in leaves of clover, lettuce, ryegrass, or rape, irrigated with water containing 2.1 mg/L MCs. However Mohamed and Al Shehri (2009) [68] reported MCs in leaves of different vegetable crops irrigated with contaminated groundwater. Peuthert et al. (2007) [69] also reported low accumulation of MCs in leaves of 11 agricultural crops and higher accumulation in roots. Another study described a positive correlation between the accumulation of MCs in lettuce and the different exposure concentrations of MCs (MC and MC-RR) [61], which is in agreement with the pattern found in this work for MC.

Regarding CYN accumulation in vegetables, there are fewer data available. Kittler et al. (2012) [70] carried out a study of CYN uptake and accumulation in kale (*Brassica oleracea* var. sabellica) and mustard (*Brassica juncea*). The authors reported significant levels of toxin in the leaves (2.71 ± 0.65 and 3.78 ± 0.47 µg/kg fw in kale and in vegetable mustard respectively) in aeroponic cultures irrigated with toxic cyanobacterial extracts (18.2 µg/L CYN). Cordeiro-Araújo et al. (2017) [19] also reported a concentration-dependent CYN bioaccumulation in lettuce, and the mean value reported after exposure to 10 μg/L CYN for 7 days was 3.78 ± 0.25 µg/kg fw. Relatively higher values were observed in the present work in lettuce and spinach, which might be due to the increased time of exposure to the toxin. 

Assuming a consumption of 40 g of vegetable per day for an adult of 60 kg (equivalent to 0.67 g/kg bw day) [19,36], the dietary intake of CYN would vary between 0.00067–0.028 µg/kg bw per day with the ingestion of contaminated lettuce leaves, and between 0.008–0.08 µg/kg bw day with the ingestion of contaminated spinach leaves. Taking into account the proposed TDI for CYN of 0.03 µg/kg per day, the consumption of spinach with the highest level of contamination would be potentially adverse to human health.

## 3. Conclusions

The contamination of vegetables and plant products raises growing concerns as a result of the increasing degradation of water resources used in irrigation and the presence of toxic cyanobacteria in irrigation waters. Despite the advances in the understanding of the effects of cyanotoxins on plants, for example, plant development and productivity, the multiple environmental factors that influence the action of cyanotoxins and the response of plants to cyanotoxins makes risk analysis extremely difficult, needing more scientific knowledge. This work provides new evidences concerning the effects of cyanotoxins at environmental concentrations. Overall MCs and CYN, separately or in combination, at a concentration of 50 μg/L in plant’s growth medium (hydroponic conditions) were detrimental to the development of lettuce and spinach. When the same amount of toxin was present, the CYN+MC mixture showed to be more toxic then CYN alone. From this result, it may be speculated that cyanotoxins can act synergistically increasing the toxic potential of the water. Moreover, for purposes of toxicity assessment of environmental and irrigation waters, the presence of different cyanotoxins in the water should be monitored and their potential synergistic effects taken into account. On the one hand, MC concentrations in irrigation waters may raise more concerns due to the detrimental effects on plant growth. On the other hand, CYN is assimilated by the plant in greater amount than MC, leading to the conclusion that the use of water contaminated with this toxin is particularly concerning with regard to food safety and human exposure. Moreover some crops could be more sensitive to this toxin than others, and accumulate more of this toxin in the tissues. Given the risks identified with the exposure to CYN and/or MC at 50 μg/L, we suggest that concentrations lower than 50 μg/L should be considered for establishing the regulatory limits of cyanotoxins in irrigation waters.

Additionally, the diverse effects found in the mineral content of cyanotoxins exposed plants should be further studied because might have significant implications on plant development as well as in plant nutritional value.

## 4. Materials and Methods

### 4.1. Biological Material

*Microcystis aeruginosa* (LEGE 91094) and *Chrysosporum ovalisporum* (LEGE X-001) were grown as previously described by Campos et al. (2013) [71] in the Interdisciplinary Centre of Marine and Environmental Research, CIIMAR (Porto, Portugal). The lyophilized material was stored at room temperature in the dark for toxin extraction.

*Spinacia oleracea* (spinach) and *Lactuca sativa* (lettuce) were purchased as sprouts in a local market (Porto, Portugal). Plants were washed with deionized water in order to remove all remaining soil present in roots. Then, plants were cultivated in a hydroponic system according to Freitas et al. (2015) [30]. Briefly, plants were placed into 100 mL opaque glass jars, randomly distributed in groups and acclimated in Jensen culture medium [72] during a week (14–10 h, light-dark period and 21 ± 1 °C). After that, the plants were utilized for the exposure experiments.

### 4.2. Cyanobacterial Crude Extracts and Quantification of MC and CYN

MC and CYN extractions from cultures of *M. aeruginosa* and *C. ovalisporum*, respectively, were performed following the procedures described by Pinheiro et al. (2013) [73] and Welker et al. (2002) [74]. Analysis by high-performance liquid chromatography photodiode array detection (HPLC-PDA) showed a mean content of 0.2 mg MC/g and 2.9 mg CYN/g of lyophilized material, with retention times of 9.75 min (MC) and 6.305 min (CYN).

### 4.3. Exposure Experiments

After acclimation, five experimental groups for lettuce and seven for spinach were outlined comprehending the plants irrigated with non-contaminated water (control group), plants irrigated either with *M. aeruginosa* or *C. ovalisporum* extracts containing environmentally realistic toxin concentrations (10 and 50 µg/L MC or CYN), and plants irrigated with a mixture of both cyanobacterial extracts (toxin concentration of 5 µg/L MC + 5 µg/L CYN and 25 µg/L MC + 25 µg/L CYN). Eight replicates of each treatment were performed. In order to simplify the description, the water with *M. aeruginosa* extracts will be referred to as “MC-contaminated water” and the water with *C. ovalisporum* extracts will be referred to as “CYN-contaminated water” throughout the manuscript. The toxin concentration in the cyanobacterial extract was always quantified before preparing the artificially contaminated water to certify that the correct concentrations of MC or CYN in the irrigation water were present.

The plant culture media and toxins were replaced three times a week for 21 days. At the end of the experiments, plants were washed with deionized water and underwent different pre-treatment depending on the analysis.

### 4.4. Physiological Parameters: Plant Fresh Weight and Photosynthetic Capacity

Immediately after harvesting the plants, leaves and roots were separated and weighted (fresh weight, fw), and stored at –80 °C for further analysis. Plant growth was expressed as the mean fresh weight (fw) ± SD from eight plants (n = 8) per treatment.

Photosynthetic capacity was determined through pulse amplitude modulation (PAM) fluorometry, with PAM 2000 (Walz, Effeltrich, Germany) instrument according to Machado et al. (2017) [36]. Plants were first kept in dark conditions for at least 30 min and leaves subsequently illuminated with a pulse of saturating light. The fluorescence emitted was recorded by the instrument. This procedure allows measuring the maximum fluorescence yield of photosystem II (PSII)(Fv/Fm) that is directly related with the functional state of the PSII protein complex and the photosynthetic efficiency of the plants [59].

### 4.5. Mineral Content

Determination of mineral content was developed following Freitas et al. (2015) [30]. Briefly, freeze-dried samples (leaves and roots from spinach and lettuce) were digested by microwave-assisted acid digestion using a MLS 1200 Mega system (Milestone, Sorisole, Italy). Sample solutions were then analyzed by flame atomic absorption spectroscopy (FAAS) using a 200 Analyst equipment (Perkin Elmer, Überlingen, Germany) and by inductively coupled plasma-mass spectrometry (ICP-MS) using an iCAPTM Q (Thermo Fisher Scientific, Bremen, Germany) for total metal content. Results were expressed on a dry weight (dw) basis.

### 4.6. MC Extraction and Quantification from Spinach

MC were extracted from leaves of spinach exposed to 0, 10, and 50 µg/L MC following the method described by Prieto et al. (2011) [31] with slight modifications. Lyophilized spinach leaves (0.05 g) were mixed with 10 mL of 0.1 M acetic acid and 20 mL of a 1:1 (*v*/*v*) mixture of methanol–chloroform. Then, the mixture was stirred (15 min.) and sonicated (15 min.) three times at 4 °C and then centrifuged at 3400× *g*. When the supernatant was collected, the extracts were purified according to Guzmán-Guillén et al. (2011) [75], using Oasis HLB cartridges (500 mg/6 mL, Waters, Mildford, MA, USA). Chromatographic separation was performed using a UPLC Acquity (Waters, Mildford, MA, USA) coupled to a Xevo TQ-S micro (Waters, Mildford, MA, USA) consisting of a triple quadrupole mass spectrometer equipped with an electrospray ion source operated in positive mode. UPLC analyses were performed as described by Díez-Quijada et al. (2018) [18]. The transitions employed for MC are 996.5/135.0, 996.5/213.1 and 996.5/996.5.

### 4.7. CYN Extraction and Quantification from Spinach and Lettuce

The extraction and purification of CYN from plant materials was performed according to Prieto et al. (2018) [64]. Briefly, lyophilized biomass was extracted with 6 mL of 10% acetic acid, homogenized by ultraturrax, sonicated (15 min) and stirred (15 min). Then, the mixture was centrifuged for 15 min (12,000× *g*) and the supernatant was collected and purified. The purified CYN fractions were concentrated (solvent evaporation) and resuspended in 1 mL Milli-Q water prior to its UPLC/MS-MS analysis, following the method described by Prieto et al. (2018) [64].

### 4.8. CYN/MC MIX Simultaneous Extraction and Quantification from Spinach and Lettuce

The determination of toxins in CYN/MC mixtures was performed following the method described by Diez-Quijada et al. (2018) [18]. Lyophilized leaves and roots were extracted with 6 mL of 80% methanol, ultraturrax (1 min), sonicated (15 min) and stirred (15 min). Then, the mixture was centrifugated (3400× *g*, 15 min) and the supernatant collected for clean-up. An assembly of a C_18_ Bakerbond cartridge (500 mg, 6 mL, Dicsa, Andalucia, Spain) and a BOND ELUT Carbon cartridge (Agilent Technologies, Amstelveen, The Netherlands) was employed. After adjusting the supernatant to pH 11, the following reagents were passed through the assembled cartridges: 6 mL DCM, 6 mL 100% MeOH, 6 mL H_2_O (pH 11) and sample (pH 11): then, cartridges were dried for 5 min and eluted with 10 mL DCM/MeOH (40/60) + 0.5 formic acid. Then, the extracts were evaporated to dryness and resuspended in 1 mL 20% MeOH for its analysis by UPLC-MS/MS, according to Diez-Quijada et al. (2018) [18].

### 4.9. Statistical Analysis

Leaf weight, root weight and photosynthetic rate data from the two plant species were analyzed with General Linear Models, in which plant dry weight, root dry weight and photosynthetic rate were used as response variables and the cyanobacterial treatment as factor. Assumptions of residual normality and homoscedasticity were tested with Shapiro-Wilks and Levene tests, respectively. If any of the assumptions was not met, data were transformed using the Box-Cox transformation. Tukey post-hoc tests between factor levels were performed when due. All these analyses were performed with R software, with functions from base, stats, MASS, car and multcomp packages.

Spinach and lettuce ionome (the abundances of chemical elements on individual plants) is a kind of quantitative data from a series of elements that constitute parts of a whole, usually given in proportions. This kind of data are considered in Statistics as Compositional data. These data usually involve redundancy and multiple correlations of spurious nature that make inefficient the use of most standard statistical techniques, and hence, need specific procedures for its analysis, that we describe here below.

A sample space of compositional data, such as the data of plants ionome, is defined by *S^D^*, a positive vector of *D* components adding up to a constant *k*, such as 100% or some other constant sum. The “closure” operator *C* normalizes the contained vector as follows:

$$S^{D}=C\left(c_{1},c_{2},\ldots ,c_{D}\right)=[\frac{c_{1}K}{\sum_{i=1}^{D}c_{i}},\frac{c_{2}K}{\sum_{i=1}^{D}c_{i}},\ldots ,\frac{c_{D}K}{\sum_{i=1}^{D}c_{i}}]$$

where *k* is the unit of measurement and *c_i_* is the *i*th part of a composition containing *D*-parts [76]. Due to the nature of the sample space, independence hypotheses must clearly take different forms from those associated with *S_D_*. Thus, the sample space should be divided into non-overlapping subcompositions where each subcomposition can be interpreted independently. In order to do this, the ionome data were recalculated to sum up to a constant value (1 in our case) per individual plant.

For the elimination of redundancy and spurious correlations, as well as getting data distribution closer to normality, it is necessary to apply the isometric log-ratio (*ilr*) transformation. First, new orthogonal variables were created, through the combination of those already existing, thus eliminating linear dependency. This task was performed by constructing a sequential binary partition (SBP) from the whole ionome data. This technique consists in combining the elements in balanced ratios that are orthogonal. From n elements in the ionome matrix, n—1 orthogonal balances would be obtained. The orthogonality is determined by assigning orthogonal coefficients to *ilr* transformed balances in all possible subcompositions of the data set (see Parent et al. 2013) [77]. Those orthogonal balances are non-redundant and scale-invariant. This task was performed with the functions *acomp*, *gsi.merge2signary* and *gsi.buildilrBase*, from the *R* package *compositions*. The *ilr* transformation has the following formula:

$$ilr_{j}=\sqrt{\frac{r_{j}\;s_{j}}{r_{j}+s_{j}}}\ln\frac{g\left(c+{\;}_{j}\right)}{g\left(c{-}_{\;j}\right)}$$

where *ilrj* is each of the isometric log-ratios, *r_j_* and *s_j_* are those elements with positive and negative orthogonal coefficients, respectively, that integrate the *j*th balance. *g* (*c + _j_*) and g (*c − _j_*) are the geometric means of those elements with positive and negative coefficients, respectively.

## Figures and Tables

**Table 1:** Levels of MC and CYN in tissues of lettuce and spinach exposed to the 2 cyanotoxins, measured by UPLC-MS/MS as described previously [18]. These results were first published in Díez-Quijada et al. (2018) [18]. < LOD—below the detection limit of the method (0.06 ng MC/g fw and 0.07 ng CYN/g fw); nd—not determined. Number of sample replicates (n = 3). Concentrations of exposure were: 10 µg/L (CYN10 and MC10) and 50 µg/L (CYN50 and MC50) and CYN/MC mixture at the concentrations of 5 + 5 µg/L (MIX10) and 25 + 25 µg/L (MIX50), respectively, for 21 days.

Levels of MC and CYN in Tissues of Lettuce and Spinach		CYN10	CYN50	MC10	MC50	MIX10	MIX50	MIX10	MIX50
		CYN	CYN	MC	MC	MC	MC	CYN	CYN
		(µg/kg fw)	(µg/kg fw)	(µg/kg fw)	(µg/kg fw)	(µg/kg fw)	(µg/kg fw)	(µg/kg fw)	(µg/kg fw)
**Lettuce**	leaves	2.4 ± 0.89	9.4 ± 2.38	nd	nd	< LOD	< LOD	10.00 ± 2.40	41.92 ± 6.37
	roots	nd	nd	nd	nd	0.22 ± 0.08	1.10 ± 0.25	34.23 ± 11.58	110.00 ± 33.29
**Spinach**	leaves	9.52 ± 3.56	36.97 ± 10.25	< LOD	< LOD	< LOD	< LOD	12.57 ± 4.22	119.69 ± 43.93
	roots	nd	nd	nd	nd	0.53 ± 0.32	1.31 ± 0.14	39.16 ± 31.30	24.00 ± 3.76
